# Contrasting multitaxon responses to climate change in Mediterranean mountains

**DOI:** 10.1038/s41598-021-83866-x

**Published:** 2021-02-24

**Authors:** Luca Di Nuzzo, Chiara Vallese, Renato Benesperi, Paolo Giordani, Alessandro Chiarucci, Valter Di Cecco, Luciano Di Martino, Michele Di Musciano, Gabriele Gheza, Chiara Lelli, Daniel Spitale, Juri Nascimbene

**Affiliations:** 1 Dipartimento di Biologia, Università di Firenze, Via la Pira 4, 50121 Florence, Italy; 2 Biodiversity and Macroecology Group, Department of Biological, Geological and Environmental Sciences, Alma Mater Studiorum - University of Bologna, Via Irnerio 42, 40126 Bologna, Italy; 3 Dipartimento di Farmacia, Università di Genova, viale Cembrano, 4, 16148 Genoa, Italy; 4 Parco Nazionale della Majella, Via Badia, 28, 67039 Sulmona, Italy; 5 Department of Life, Health and Environmental Sciences, University of L’Aquila, Piazzale Salvatore Tommasi 1, 67100 L’Aquila, Italy; 6 Museo di Scienze Naturali Dell’Alto Adige, Via Bottai, 1, 39100 Bolzano, Italy

**Keywords:** Climate-change ecology, Community ecology

## Abstract

We explored the influence of climatic factors on diversity patterns of multiple taxa (lichens, bryophytes, and vascular plants) along a steep elevational gradient to predict communities’ dynamics under future climate change scenarios in Mediterranean regions. We analysed (1) species richness patterns in terms of heat-adapted, intermediate, and cold-adapted species; (2) pairwise beta-diversity patterns, also accounting for its two different components, species replacement and richness difference; (3) the influence of climatic variables on species functional traits. Species richness is influenced by different factors between three taxonomic groups, while beta diversity differs mainly between plants and cryptogams. Functional traits are influenced by different factors in each taxonomic group. On the basis of our observations, poikilohydric cryptogams could be more impacted by climate change than vascular plants. However, contrasting species-climate and traits-climate relationships were also found between lichens and bryophytes suggesting that each group may be sensitive to different components of climate change. Our study supports the usefulness of a multi-taxon approach coupled with a species traits analysis to better unravel the response of terrestrial communities to climate change. This would be especially relevant for lichens and bryophytes, whose response to climate change is still poorly explored.

## Introduction

Climate change is already harming biodiversity worldwide becoming one of the major causes of species extinctions in the next decades^1,2^. Organisms react to climate change through a wide spectrum of responses^3^. For example, through changes in phenology^4^, physiology^3^, community structure and distribution ranges^5^. Among these, latitudinal and altitudinal range shifts are probably the most threatening for ecosystem biodiversity and functioning^6^. In particular, altitudinal shifts could cause major diversity loss among plants and cryptogams (i.e. lichens and bryophytes) as mountains host several endemic and specialist, cold-adapted species that meet their altitudinal optimum above the tree-line^7,8^. As temperature rises, their suitable habitat is moving upward, but due to topography and land cover constrains this mainly results in an altitudinal range loss^9,10^. At the same time, warm-adapted (thermophilous) and more competitive species are expanding upward^8^. Thus, in addition to range loss, high-altitude species will face an increasing competition in their lower range limit. This could cause a decline or local extinctions of cold-adapted species in the future^8,11^, resulting in compositional shifts of local communities.

Southern mountains of the northern hemisphere (as those in the Mediterranean area) often host species’ range-edge populations that are expected to respond differently from core populations^12^. In fact, in these range-edge environments climate is considered harsher than species’ core environments. Thus, from one hand it is expected that these populations have evolved local adaptations to persist^12,13^ and become more tolerant to climatic changes. On the other hand, they will likely face greater climatic changes than core populations. Hence, the study of these edge environments is fundamental to develop a reliable model of how communities will be affected by climate change.

Altitudinal gradients have proved to be of crucial importance to explore the effect of climatic-induced changes on biodiversity^14,15^. Along altitudinal gradients, abiotic factors vary in a relatively short distance, simulating wider climatic and ecological succession in time. In particular, temperature is negatively correlated with elevation, although this trend could have low variation in relation to latitude and topography^14,16^. Moreover, other abiotic factors such as precipitation and seasonality also vary with altitude even if their relationship with elevation is more complex^14^. In fact, these factors are also influenced by topography and distance from the sea. As instance, while some mountains could be dry at base and humid in uppermost part others have higher precipitation in a mid-altitude zone^16^.

Using a multi-taxon approach coupled with a species traits analysis along elevational gradients is a promising approach to better elucidate complex community diversity patterns^17^. Considering evolutionary distant taxa provides a broad insight into the effects of climate on biodiversity, as related species could preserve their ecological niche (niche conservatism) and thus respond similarly to climatic changes^17,18^. However, it is expected that abiotic factors influence biological communities affecting the species through their functional traits. In this perspective, functional traits can be directly related with environmental changes^19^. Despite the importance of simultaneously considering community dynamics and trait-mediated responses of different organism groups, multi-taxon studies along environmental gradients are still scanty^17^, especially those including cryptogams (e.g.^20,21^).

In this study, we explored the influence of climatic factors on diversity patterns of lichens, bryophytes and vascular plants along a steep elevational gradient in the Majella Massif (Abruzzo, Italy) to predict communities’ dynamics under future climate change scenarios. The Majella Massif is a unique site to test the effects of climate on cryptogam and plant communities since it is the southernmost Mediterranean mountain with an alpine and subalpine belt in Italy, hosting several artic-alpine species that are at their southernmost distribution limit and several endemics^22,23^ likely impacted by climate change.

Due to different morphological traits and eco-physiological and dispersal strategies the response to climatic factors may strongly differ between cryptogams (i.e. lichens and bryophytes) and vascular plants, resulting in contrasting diversity patterns. In particular, we hypothesized that: (a) both community richness and composition patterns may differ between cryptogams and vascular plants and that (b) these patterns are mediated by particular functional traits. For example, lichens and bryophytes are slow-growing poikilohydric organisms, in which water content varies with ambient moisture. Almost all lichen and bryophyte species are desiccation tolerant, i.e. they can lose most of their cell water without dying and resume their function when rehydrated^24^. This trait allows them to grow during wet conditions and suspend their metabolism in dry periods^25^. Thus, these poikilohydric organisms are expected to be more affected by climate than vascular plants, since their physiology is strongly related to climatic factors such as temperature and precipitation. To test these hypotheses we analyzed along a climatic/elevational gradient (1) species richness patterns, also accounting for contrasting temperature-affinities groups, as in the case of thermophilous, and cold-adapted species (*Generalized Linear Mixed Models*); (2) pairwise beta-diversity patterns, also accounting for its two different components, species replacement and richness difference (*Partitioning of β-diversity*) that allow to disentangle the mechanisms underlying community assembly^26^; (3) the influence of climatic variables on species functional traits (*Fourth corner analysis*).

## Materials and methods

### Study area

The study was carried out in the Majella National Park (Abruzzo, Italy) in Central Apennines. This area is characterized by meso-cenozoic organogenic limestones mainly derived from sedimentation in shallow water in a Mesozoic-Tertiary platform. The Majella massif is orientated along a N-S direction, with its highest summit in Monte Amaro (2793 m). The area selected for our study lies along the massif ridge between 42° 00′ 23″ N (Blockhaus) and 42° 09′ 41″ N (Guado di Coccia). Due to a complex geological history and the influence of karst, glacial and fluvial processes, the massif presents a variety of different landforms. The summit is flattened by a periglacial altipiano, bordered by deep and incised valleys, representing a unique situation in the Apennine with more than 24 km^2^ above 2400 m. The closest and highest weather station is in Campo Imperatore (2125 m) with 3.6 °C mean annual temperature and 1613 mm annual precipitation. In the lowest belts, the vegetation is dominated by almost monospecific *Fagus sylvatica* L. subsp. *sylvatica* stands. The dominant vegetation above the timberline, until 2100–2200 m, is composed by shrublands with *Juniperus communis* L. and, in the northern part, with *Pinus mugo* Turra^27^. This vegetation shifts to high elevation grasslands in the subalpine and alpine belts. Furthermore, the northern part of the park is the only case in the Apennines where *P. mugo* dominates the transition at the timberline, a typical alpine condition^28^. Patches dominated by *P. mugo* are in expansion mainly due to climate change and the abandonment of pasturing since 1950s, which only remains at low intensity in the lowest part of the massif^28^.

### Sampling design, species identification and nomenclature

**Figure 1:**
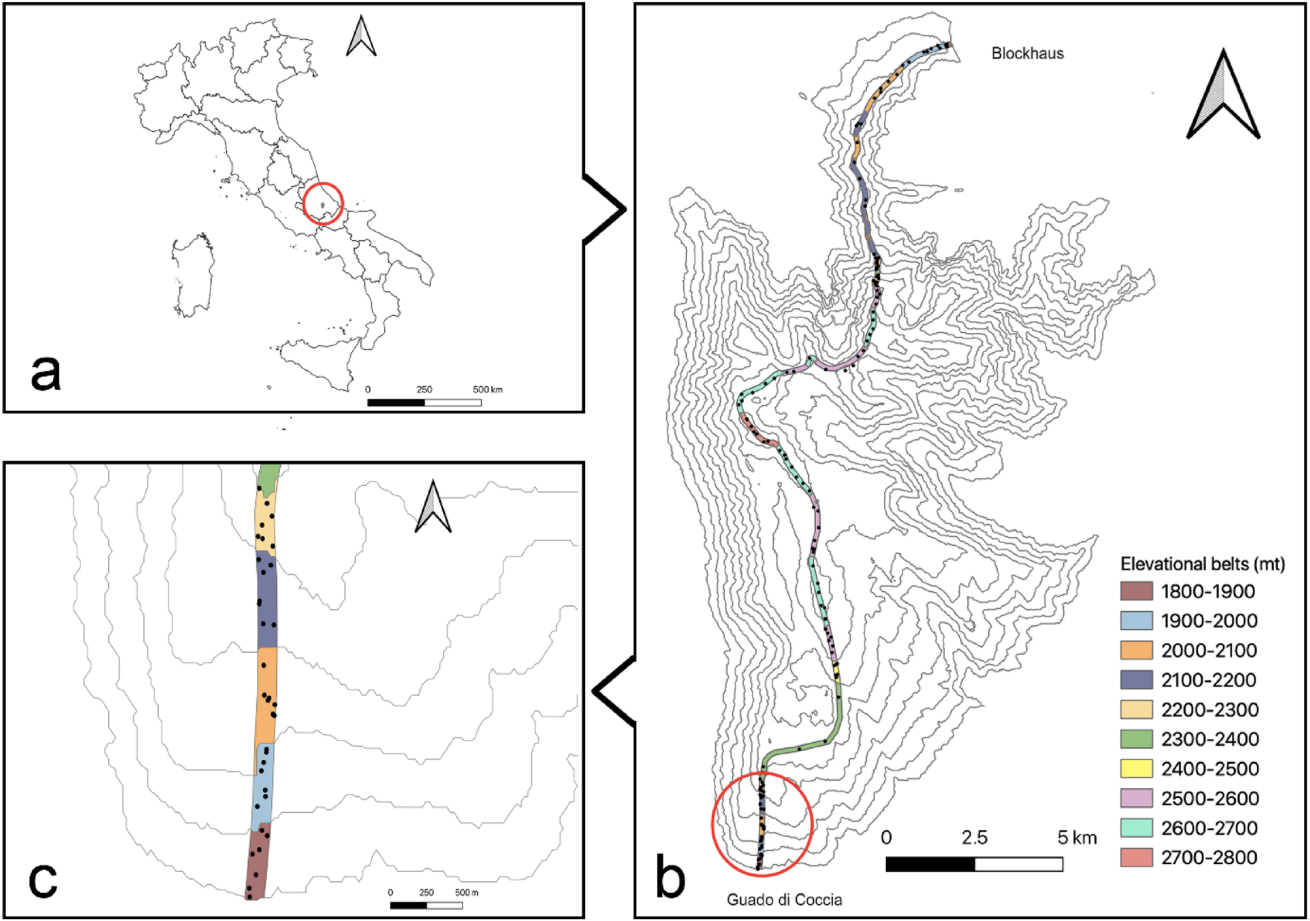
Location of the study area (**a**) and conceptualization of the sampling design (**b**,**c**). The elevational transect was split into ten 100 m belts (**b**). In each belt, 7 plots of 100 × 100 cm were randomly selected (**c**). Figure was produced using the open-source software QGIS 3.10.12 (https://www.qgis.org) and assembled using Adobe Photoshop CC 2018. In particular, figure (**a**) is based on the shapefiles freely available on the ISTAT (Istituto nazionale di statistica) website: https://www.istat.it/it/archivio/222527. The contour line in figure (**b**) were calculated using Contour function available in QGIS 3.10 and using a 10 m resolution DTM freely available on INGV Pisa (Istituto nazionale di geofisica e vulcanologia – Sezione di Pisa) website: http://tinitaly.pi.ingv.it/Download_Area2.html.

The elevational transect was designed along the main ridge of the Majella Massif, covering both north and south faces above timberline. The transect had an overall length of 14 km and a width of 100 m, reaching the highest altitude at Monte Amaro peak. The transect started above the timberline and continued to the top, ranging from 1800–1900 to 2700–2793 m. We split the transect into 100 m belts in which we randomly selected 7 plots of 100 × 100 cm. The belts spanning from 2500–2600 and 2600–2700 m had disjunctions due to heterogeneous pattern of the slope. In these two cases, we selected more than 7 plots. The final dataset contained, therefore, 154 plots. Within each plot, we recorded the occurrence of all lichen, bryophyte and vascular plant species (Fig. 1).

All lichens and bryophytes were collected for laboratory identification since identification in the field was not possible. When necessary, specimens were analysed with dissecting and standard light microscopes, and routine chemical spots for lichens^29^. Furthermore, we performed standardized thin-layer chromatography for sterile lichen specimens^29^. Critical specimens were sent to specialists to provide correct identification. Vascular plant species were identified mainly in the field. In case of specimens without critical characters, the identification was stopped at the genus level.

Nomenclature followed Nimis and Martellos^30^ for lichens, Ros et al.^31^ for bryophytes, and Conti et al.^22^ for vascular plants. All lichen and bryophyte specimens (c. 800) were stored in the personal herbarium of the senior author (JN).

### Functional traits

We characterized every species of each taxonomic group by a suite of functional traits available in the literature and that are known to respond to environmental gradients^32,33^ (see Supplementary Tables S1, S2, and S3 online). For lichens, we used growth form, photobiont type and reproductive strategy following Nimis and Martellos^30^. In general, photobiont type is related to water availability, amount of light and temperature, growth form is related to water uptake and temperature, and reproductive strategy is related to dispersal and habitat stability. For bryophytes, we characterized each species by shoots length and simplified growth forms according to Hill et al.^34^. In mosses and liverworts shoots length and growth form are important features in regulating water retention and reducing air resistance. For example, bryophytes with loose shoots are more subjected to lose water, whereas cushion forms are highly adapted to water retention^35^. Finally, for vascular plants we used the maximum height and simplified life forms following Pignatti^36^ that are both related to the adaptation to climatic factors^37^.

### Temperature-affinities groups

The species of the three taxonomic groups were assigned, on the basis of their ecological affinity to temperature, to three categories: (a) *cryophilous,* including cold-adapted and strictly arctic-alpine species, (b) *intermediate*, including species adapted to a wide climatic (mainly temperature) gradient but with their optimum under mesic conditions, and (c) *thermophilous*, including species adapted to warm conditions. The assignment of lichen species to the different *temperature-affinities groups* was based on the ecological and distributional information available in Nimis and Martellos^30^. In particular, for each species we combined the elevational index, expressed according with an ordinal scale of 5 values, along with frequency of observation in different ecoregions of Italy. Species with a wide altitudinal range and higher presence in ecoregions associated with warmer climate were assigned to *thermophilous.* By contrast, species with a wide altitudinal range but found more often in lower mountain areas were considered as *intermediate.* Those lichen species with an altitudinal range restricted to higher elevation and found more in alpine ad subalpine ecoregion were assigned to *cryophilous*. For bryophytes, we followed the Flora Indicativa^38^. We categorized species with T index = 1 as *cryophilous*, T index = 2–3 as *intermediate* and T index = 4 as *thermophilous*. For vascular plants, we used the T index of Ellenberg in Pignatti^39^. Species with T index = 1–2 were considered as *cryophilous*, those with T index = 3–4 as *intermediate*, and those with T index > 4 and with X (no preference) as *thermophilous*.

### Climatic variables

For each sampling plot, we obtained a set of 19 climatic variables from CHELSA database^40^. This dataset provides 11 temperature and 8 precipitation variables with a resolution of 1km^2^. Due to their grain, they are very useful for large-scale studies, but they provide more limited information at small spatial scales^41^. Thus, all the variables were downscaled to a resolution suitable for our study. For temperature-related variables, we downscaled the variables fitting a generalized linear model (GLM)^42^ with the temperature variables as covariate and altitude and northness as independent variable, this latter extracted from 20 m resolution Digital Elevation Model (DEM). In this way, we spatialized each temperature variable to 20 m/pixel resolution. In the case of precipitation-related variables, since they did not have a clear relationship with topographic variables, we used linear interpolation of CHELSA rasters to obtain a 20 m/pixel resolution.

### Statistical analyses

All statistical analyses were performed in R version 3.6.2^43^.

For each plot we calculated the species richness for each taxonomic group (lichens, bryophytes, and plants). Due to the low occurrence of liverworts in our dataset, we analysed them together with mosses. Furthermore, species richness was calculated for each temperature-affinities group within taxonomic groups.

#### Generalized linear mixed models

To investigate the influence of the climatic variables on species richness of the three taxonomic groups we fitted generalized linear mixed models^44^ using the glmmADMB package^45^. The altitudinal belt was used as random factor to account for the spatial dependence of the mountain slopes. As species richness of lichens and bryophytes was overdispersed, we used a negative binomial error distribution. The possibility to add a zero-inflation component was accepted for lichens and discarded for bryophytes after comparing models through Akaike Information Criterion (AIC). For plants we used a Gaussian error distribution. In addition, we fitted the same models for the species richness of the three temperature-affinities groups of each taxon separately as dependent variables, keeping the same error distribution as before, i.e. binomial for bryophytes and lichens, and Gaussian for plants. In order to reduce collinearity, we retained only those variables that were not highly correlated (pairwise Pearson correlation <|0.7|). Moreover, considering the ecology of considered taxonomic groups we choose those variables that were more likely to influence species of each taxon. Thus, as explanatory variables we selected: (1) mean annual temperature (BIO1), (2) annual precipitation (BIO12), (3) temperature seasonality (BIO4) and (4) precipitation seasonality (BIO15). Temperature seasonality is the standard deviations of the monthly mean temperature representing the temperature variation over a year. Precipitation seasonality is the coefficient of variation of monthly precipitation which is a measure of the variation in monthly precipitation along the year^40^. To provide a better parametrization of the variables and due to the exploratory nature of the study, we used a multimodel inference within an information-theoretic approach^46^. All possible models derived from the combination of the parameters described above were fitted and ranked using the corrected Akaike Information Criterion (AICc). AICc is proportional to the maximized log-likelihood, sample size and number of parameters of the models. AICc gives information about the relative model fit. All possible models are individually compared with the ‘best’ model, i.e. with the lowest AICc. The delta AICc (ΔAIC), which informs on the relative support for the candidate model, is calculated as ΔAICc_i_ = AICc_i_ − AICc_i_ min. We followed an AIC-based model selection procedure, considering equivalent all models with ΔAICc < 4^47^. Akaike weight (wi) was calculated for each selected model in order to assess their relative plausibility. This value, that ranges between 0 and 1, is a measure of how likely a model would be selected if the sampling of the data would be repeated. We also calculated the sum of weight (SW) for each variable, which is the sum of the weights of each model where the variable is present and informs about the probability for each variable to be included in the best model. Thus, SW provides information about the relative variable importance (RVI)^48^. To perform multimodel inference analysis we used MuMIn package^49^.

#### Partitioning of β-diversity

To estimate the relative importance of beta diversity components along the climatic/elevational gradient, we decomposed the overall beta diversity following Carvalho^50^ and using the Sørensen index. This approach is based on the idea that the total beta diversity (βtot) is generated by two processes, replacement of species (βrepl) and richness difference, i.e. the difference in species richness between sites (βrich). Following Legendre^26^ we calculated the averaged partition values on the whole dataset for the three taxonomic groups separately through *beta.multi* function in BAT package^51^.

To test the influence of climatic factors on the two components of β-diversity for each taxonomic group we performed a PERMANOVA^52^ with 999 permutations through the function *adonis* of vegan R package^53^.

#### Fourth corner analysis

To assess the influence of climatic variables on species functional traits, we performed a fourth corner analysis. This procedure allows testing the influence of environmental variables on species functional traits combining three matrices: (1) a sample units × species abundance or presence-absence matrix, (2) a sample units × environmental variables matrix and (3) a species × traits matrix. We used the model-based approach proposed by Brown et al.^54^, fitting a model with all species at the same time (presence-absence in our case) as a function of environmental variables, species traits and their interaction. This method allows to test the significance of the association between environmental variables and traits and the intensity of such association. We fitted a generalized linear model with binomial error distribution through the *traitglm* function in mvabund R package^55^. The model was fitted with a least absolute shrinkage and selection operator (LASSO) penalty that simplify the model switching all the terms that do not explain any variation to zero^54^.

## Results

In the 154 plots we found 270 taxa (75 lichens, 44 mosses, 3 liverworts and 148 plants). Mean plot species richness was 18 (minimum 4, maximum 37), with the lowest value for bryophytes (2 species), followed by lichens (3 species), and plants (12 species). The range of species per plot varied from 0 to 19 for lichens, from 0 to 11 for bryophytes and from 0 to 23 for plants. Details on the number of species assigned to each temperature-affinities group, and categorical functional trait are reported in Table 1.

**Table 1:** Number of species in each taxonomic group, temperature-affinities group, and categorical functional traits.

	Lichens	Bryophytes	Plants
Overall	75	47	148
**Temperature-affinities groups**			
Cryophilous	30	13	33
Intermediate	28	17	59
Thermophilous	17	17	56
**Growth form/life forms**			
Crustose	38	–	–
Squamulose	17	–	–
Foliose	12	–	–
Fruticose	8	–	–
Tuft	–	12	–
Cushions	–	2	–
Turf	–	16	–
Mat	–	13	–
Weft	–	4	–
Hemicryptophyte	–	–	90
Geophytes	–	–	7
Chamaephytes	–	–	39
Phanerophytes	–	–	3
Therophytes	–	–	9
**Photobiont**			
Chlorococcoid	63	–	–
Cyanobacteria	12	–	–
**Reproduction**			
Asexual	22	–	–
Sexual	53	–	–

Temperature climatic variables are related with altitude. Mean temperature decreased linearly along the altitudinal gradient. Similarly, temperature seasonality increased in the upper parts of the transect, reaching higher values in southern face slopes. Annual precipitation increased slightly in the northern slopes. For what concern precipitation seasonality, this factor has no clear pattern along the transect.

### Generalized linear mixed models

We found contrasting species-climate relationships among the three taxonomic groups and among temperature-affinities groups (Table 2). Mean temperature and annual precipitation were the major determinants of lichen richness, which increased in colder sites with higher annual precipitation. In contrast, mean temperature and precipitation seasonality mainly influenced bryophyte richness, which increased in sites with lower seasonality. Finally, sites with higher mean annual precipitation had a higher plant species richness.

**Table 2:** Results of generalized linear mixed models.

	Mean temperature	Temperature seasonality	Annual precipitation	Precipitation seasonality
*Lichen species Richness*				
Σw_i_	**1**	0.31	**1**	0.26
Averaged parameters	**− 0.1760**	**− **0.0018	**0.0179**	0.0029
*Bryophyte species richness*				
Σw_i_	0.32	0.68	0.58	**0.92**
Averaged parameters	**− **0.0131	**− **0.0125	0.0033	**− 0.0922**
*Plant species richness*				
Σw_i_	0.27	0.52	**1**	0.31
Averaged parameters	0.0208	0.0311	**0.0486**	0.0476
*Cryophilous lichens*				
Σw_i_	**1**	0.48	**1**	0.59
Averaged parameters	**− 0.3934**	**− **0.0086	**0.0173**	0.0572
*Intermediate lichens*				
Σw_i_	0.34	0.21	1	0.2
Averaged parameters	**− **0.0202	**− **0.0005	**0.0149**	**− **0.0005
*Thermophilous lichens*				
Σw_i_	0.39	0.16	**0.95**	0.34
Averaged parameters	**− **0.0379	0.0021	**0.0178**	**− **0.0221
*Cryophilous bryophytes*				
Σw_i_	**1**	**1**	0.58	**1**
Averaged parameters	**− 0.1858**	**− 0.0383**	0.0055	**− 0.2204**
*Intermediate bryophytes*				
Σw_i_	0.4	**0.73**	0.29	0.67
Averaged parameters	**− **0.0362	**− 0.0266**	0.0011	**− **0.0801
*Thermophilous bryophytes*				
Σw_i_	0.27	0.4	0.63	0.44
Averaged parameters	0.0070	**− **0.0042	0.0040	**− **0.0224
*Cryophilous plants*				
Σw_i_	**1**	**1**	0.32	0.28
Averaged parameters	**− 1.1539**	**− 0.0470**	0.0017	**− **0.0098
*Intermediate plants*				
Σw_i_	0.31	**0.94**	**1**	0.27
Averaged parameters	0.0633	**0.0524**	**0.0339**	0.0245
*Thermophilous plants*				
Σw_i_	**1**	**0.82**	0.75	0.34
Averaged parameters	**1.1081**	**0.0373**	0.0135	0.0327

Σwi are the sum of Akaike weights, which can vary between 0 and 1. Variables with values close to one are more supported by the multi-model inference analysis. Value greater than 0.7 are shown in bold. Parameters shown are the averaged parameters for the subset models with ΔAICc < 4.

Cryophilous lichen richness decreased with increasing mean temperature, while intermediate and thermophilous lichen richness were not affected. Temperature seasonality did not affect any of the lichen temperature-affinities groups. Higher annual precipitation enhanced lichen richness in all temperature-affinities groups, by contrast precipitation seasonality had no effect. Increasing mean temperatures decreased cryophilous bryophyte richness, but did not affect intermediate and thermophilous richness. Sites with higher temperature seasonality had lower cryophilous and intermediate bryophyte richness, while thermophilous bryophyte richness had no trend. No relationship was detected with annual precipitation for all bryophyte temperature-affinities groups. Precipitation seasonality did not affect intermediate and thermophilous bryophyte richness. However, sites with higher precipitation seasonality had lower cryophilous bryophyte richness. Cryophilous plant richness decreased with increasing mean temperature, conversely thermophilous plant richness increase with increasing mean temperature. Temperature seasonality had different effect on the different plant temperature-affinities groups. In fact, while cryophilous plant richness decreased with higher temperature seasonality, intermediate and thermophilous plant richness increased with higher seasonality. Concerning precipitation factors, only intermediate plant richness increased in sites with higher annual precipitation while the other two groups are not affected. In the end, precipitation seasonality did not affect any of plant temperature-affinities groups.

### Partitioning of beta-diversity

Patterns of Beta-diversity components along the climatic/elevational gradient differed between cryptogams and vascular plants (Table 3). In cryptogams, richness difference (βrich) was the main Beta-diversity component with comparable values between lichens and bryophytes (Table 3). Conversely, in vascular plants, total beta diversity was mainly explained by species replacement rather than by richness difference.

**Table 3:** Left half table: results of PERMANOVA on the two components of Beta-diversity.

	Mean temperature	Temperature seasonality	Annual precipitation	Precipitation seasonality	Temp:Prec	Beta	Repl	Rich	Repl %	Rich %
**Lichen**						0.850	0.295	0.555	34.65	65.35
*Richness difference*										
F	3.82	0.44	7.93	1.03	12.28					
R^2^	0.022	0.003	0.046	0.006	0.071					
*p*	**0.028**	0.608	**0.003**	0.325	**0.001**					
*Replacement*										
F	16.50	4.48	2.26	4.60	**− **8.00					
R^2^	0.099	0.027	0.014	0.028	**− **0.048					
*p*	**0.001**	**0.013**	0.182	**0.007**	1					
**Bryophyte**						0.855	0.317	0.538	37.08	62.92
*Richness difference*										
F	1.70	3.84	1.41	2.33	0.29					
R^2^	0.011	0.025	0.009	0.015	0.002					
*p*	0.165	**0.025**	0.219	**0.097**	0.771					
*Replacement*										
F	4.16	**− **0.78	**− **1.36	**− **1.26	3.27					
R^2^	0.028	**− **0.005	**− **0.009	**− **0.008	0.022					
*p*	**0.019**	0.876	0.935	0.923	0.059					
**Plant**						0.814	0.608	0.206	74.65	25.35
*Richness difference*										
F	1.24	4.05	2.84	1.05	0.48					
R^2^	0.008	0.026	0.018	0.007	0.003					
*p*	0.274	**0.02**	**0.073**	0.315	0.576					
*Replacement*										
F	41.31	4.32	5.29	9.93	6.51					
R^2^	0.193	0.020	0.025	0.046	0.030					
*p*	**0.001**	**0.001**	**0.001**	**0.001**	**0.001**					

*R*^2^ the percentage of variation explained in a model, *F* the F statistic.

Significant *p* values are reported in bold. Right half of the table: partitioning of Beta-diversity (Beta) into species replacement (βrepl) and richness difference (βrich), expressed as pure value and proportion of the total beta-diversity (%).

The influence of climatic factors on the components of beta diversity varied across taxonomic groups. The richness component was mainly influenced by annual precipitation and the interaction between mean temperature and annual precipitation in lichens. At the same time, for bryophyte richness difference was influenced by temperature seasonality and precipitation seasonality, while for plants the main factors were temperature seasonality and annual precipitation. Regarding species replacement, in lichens it was mainly influenced by mean temperature and by the seasonality of temperature and precipitation. In bryophytes this component was influenced only by mean temperature. Plant replacement was influenced by all the climatic factors but to a greater extent by mean temperature.

### Fourth corner analysis

**Figure 2:**
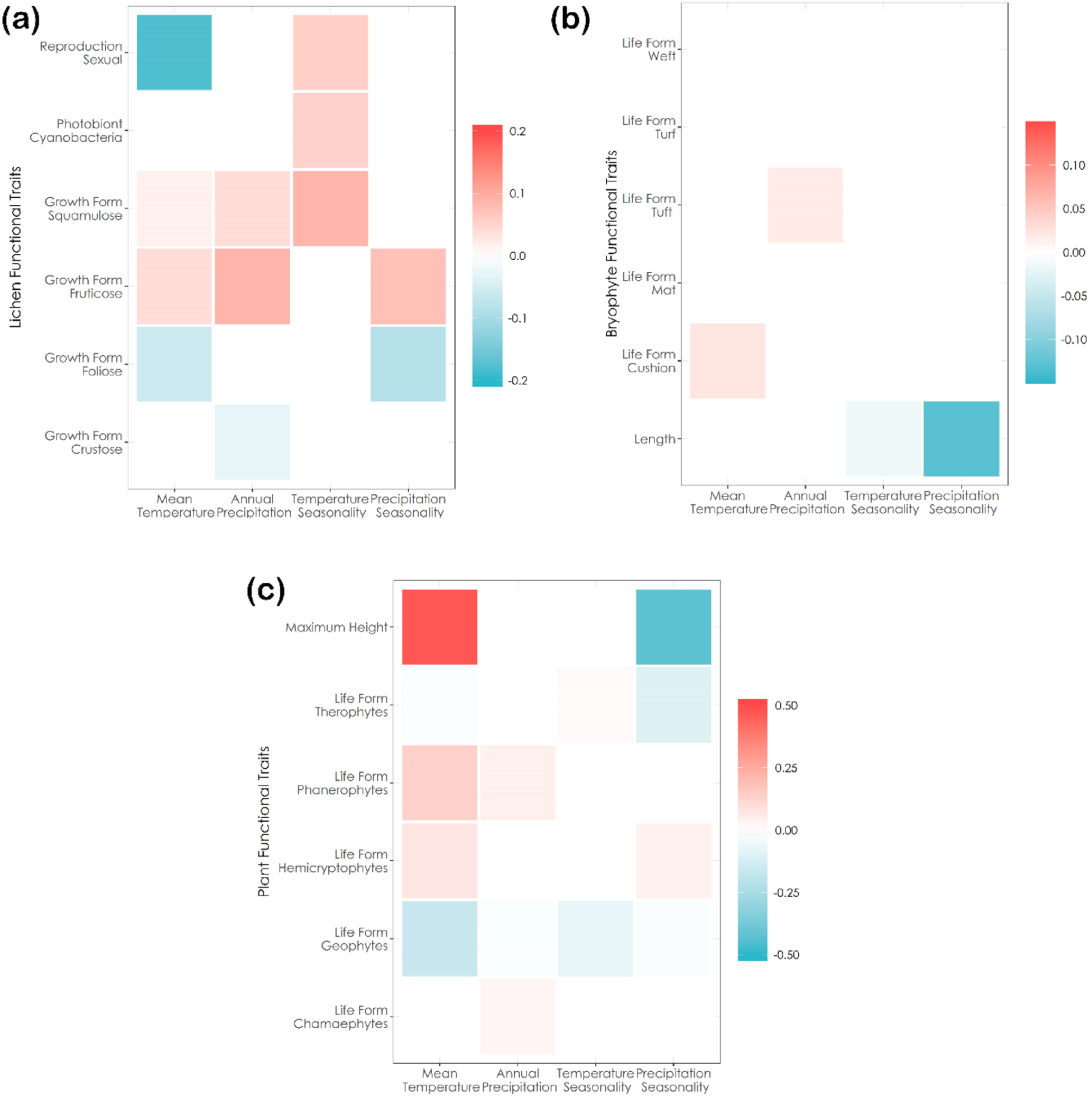
Results of the fourth corner analysis for Lichens (**a**), Bryophytes (**b**) and Plants (**c**). Boxes are coloured according to traits fourth-corner coefficients: red and blue indicate positive and negative significant trait-variable association respectively. Colour brightness indicates the strength of the association: brighter colour shows stronger association.

Climatic variables had contrasting effects on the functional traits depending on the taxonomic group considered. In lichens, climate mainly affected reproductive strategy and growth form and, to a lesser extent, the photobiont type (Fig. 2). Sexual reproduction was negatively related to mean temperature and positively related to temperature seasonality. Thus, lichens with sexual reproduction were associated to colder sites with higher temperature seasonality. Fruticose and squamulose lichens (these latter mainly include primary thalli of *Cladonia* species, thus being related to fruticose lichens) were associated with higher mean temperature and higher annual precipitation, whereas foliose species were associated with lower temperature and precipitation seasonality. Finally, crustose lichens were associated to drier sites. In bryophytes, length of the shoots was related with lower precipitation seasonality. Climatic conditions marginally influence bryophyte life forms, in fact only cushion species were marginally enhanced by increasing mean temperature. In vascular plants, plant height was increased with increasing mean temperature and decreasing precipitation seasonality. Phanerophytes and hemicryptophytes were positively related to increasing mean temperature, whereas geophytes showed an opposite trend.

## Discussion

Results of this study, based on a multi-taxon approach coupled with a species traits analysis along a climatic/elevational gradient, reveal contrasting community responses to climate change among different taxonomic groups in Mediterranean mountains. Mediterranean areas are expected to be much affected by climate change. Focusing on the area of central Apennines, studies have predicted a reduction of annual precipitation which will concentrate in winter periods. At the same time, mean temperature will increase with higher seasonal fluctuation. Moreover, extreme climatic events will increase their number through the year^56,57^.

The main differences, in terms of community richness and composition patterns, were found between cryptogams (i.e. lichens and bryophytes) and vascular plants and in all taxonomic groups are likely mediated by particular functional traits. On the basis of our observations, poikilohydric cryptogams could be more impacted by climate change than vascular plants. However, contrasting species-climate and traits-climate relationships were also found between lichens and bryophytes suggesting that each group may be sensitive to different components of climate change (e.g. variations in mean temperature/total precipitation *vs.* seasonality).

Lichen species richness had a negative relation with mean temperature and a positive one with annual precipitation. This implies that both increasing warming and aridity, that are expected to be dramatic in Mediterranean regions^58^, may strongly affect these poikilohydric organisms^59–61^ in the southernmost mountains. Our results indicate that the effect of these climatic filters should be mirrored in functional shifts of local communities mainly related to reproduction/dispersal strategy and growth form. Sexual reproduction by ascospores is usually prevalent in lichens of extreme polar and artic-alpine habitats^62^ maybe reflecting the evolutionary selection that favoured genetic variability and long range dispersal as a strategy to cope with extreme and variable climatic conditions^63^. However, under warming conditions asexual reproduction in which the fundamental components of the lichen symbiosis (e.g. the mycobiont and the photobiont) are simultaneously dispersed, may be enhanced. This likely reflects the effectiveness to counteract local extirpation as a result of a win–win tradeoff allowing both high local recruitment and establishment and moderate dispersal capability to track climate change. Also growth forms are responsive to climate, mainly reflecting a tradeoff between temperature and precipitation^15^. For example, fruticose species that currently find their most suitable conditions in the intermediate part of the elevational gradient are expected to be negatively impacted by climate change when warming is coupled with more dry conditions, as it is predicted for Mediterranean regions^58^. In contrast, more desiccation tolerant forms, as in the case of crustose or small foliose lichens, may be enhanced under warmer and dryer conditions^64^.

Bryophyte species richness seems to be more related to changes in climatic seasonality, maybe reflecting a different tolerance to drought, as compared to lichens, requiring more homogeneous conditions during the year^32^. In Mediterranean regions, increasing precipitation seasonality may negatively affect bryophyte richness likely due to exacerbate summer drought that hampers the effectiveness of water storage strategy, even in dense colonies, to sustain and prolong photosynthetic activity. This situation is reflected by the climate-traits relationship indicating that small sized-species, forming small cushions, may better cope with harsher conditions^33^. In fact, the dense structure of this life form allows to retain water for a longer period and reduce the wind turbulence, resulting in longer hydrated period^35^.

Species richness of vascular plants is mainly sensitive to annual precipitation confirming that water availability is crucial in determining plant patterns along elevational gradients (e.g.^65^). This result suggests that increasing drought stress may impact many species of high elevation ranges^56,66^. By contrast, increase in mean annual temperature can be advantageous for tall species that can shift upward their altitudinal limits. The range shifts of more resistant thermophilous species causes an increase in competition at high altitude^9,67^. These dynamics related to functional shifts of local communities mainly associated with plant size and life form can produce lost in cold habitats^9^. In particular, taller and more competitive species, as in the case of phanerophytes and hemicryptophytes, may establish and develop viable populations at higher elevation as compared to their current limit. This phenomenon could foster the lack of replacement in cryptogam communities that may be outcompeted by these plants that often tend to form dense patches.

Actually, results of Beta-diversity partitioning indicate that species loss across the elevational-climatic gradient is the main expected response of lichens and bryophytes to climate change, whereas vascular plants may be more prone to compositional turnover. Species loss in cryptogams seems to be mainly related to the loss of the cryophilous component that is stronger for lichens than for bryophytes, as indicated by the higher averaged parameter of the cryophilous species-temperature negative relationship in our model (double value). More severe climate-related shifts in lichen communities as compared to bryophytes were also found for epiphytes in the Alps^20^. In contrast to plants, the cryophilous component may be only weakly replaced by upward-moving thermophilous species (i.e. floristic thermophilization;^8^), as indicated by the non-significant effect of temperature on thermophilous species richness of both groups. As result, this could lead to a net species loss. This lack of floristic thermophilization has been already observed for terricolous bryophytes in high elevation environments^60,61^ and it seems to apply also to lichens. Reduction of biodiversity is even more dramatic in our study area. In fact, we found several species of biogeographic importance as in the case of the steppic *Circinaria hispida* (Mereschk.) A. Nordin, Savić & Tibell) or artic-alpine species such as *Allocetraria madreporiformis* (Ach.) Kärnefelt & A. Thell and *Flavocetraria nivalis* (L.) Kärnefelt & A. Thell. In general, the Majella massif hosts a large contingent of endemic plant species^22^. Artic-alpine species are adapted to colder climate, and the population on the massif are likely to be isolated from core populations. Moreover, some species like *A. madreporiformis* are closer to their south range (the population in Majella massif is the southernmost known in Europe^30^). Thus, they will be more prone to local extinction.

It is worth saying that our study deal with climatic factors and is not taking into account microscale factors, such as slope and soil moisture, which can influence the actual climatic condition experimented by these organisms.

## Conclusions

Our study supports the usefulness of a multi-taxon approach coupled with a species traits analysis to better unravel the response of terrestrial communities to climate change using elevational gradients as observational framework. In particular, considering the response of different organisms, and their interaction, to the same climatic factors may help to elucidate their vulnerability. While in the last decade information is rapidly accumulating for plants in different environmental and biogeographical ranges (e.g.^9,17,68^), including Mediterranean mountains^8,69^, cryptogams are still largely neglected, especially in southern mountains, thus hindering an exhaustive evaluation of their patterns under climate change. For example, in this study we found a congruent pattern of Beta-diversity between lichens and mosses along the climatic/elevational gradient indicating that both groups of poikilohydric organisms may be more prone to net species loss than plants. In contrast, in alpine forests^20^, thus under less climatically extreme conditions, lichens and bryophytes showed contrasting patterns, the latter being able to maintain equal species richness along an elevational-temperature gradient by species replacement. This would indicate that lichens may be strongly exposed to net species loss even in less harsh environments, while bryophytes may respond in a similar way to homohydric organisms (i.e. plants) under mesic conditions, being prone to species loss only in more extreme environments. These intriguing hypotheses would, however, require further research.

## Supplementary Information

Supplementary Tables
