# Supplementary material for: Contrasting multitaxon responses to climate change in Mediterranean mountains
Source: Sci Rep. 2021 Feb 24;11:4438. doi: 10.1038/s41598-021-83866-x (PMC7904820; doi:10.1038/s41598-021-83866-x)
Supplement: Supplementary file 1 — Supplementary Tables [file 41598_2021_83866_MOESM1_ESM.docx]

# Supplementary materials

**Contrasting multitaxon responses to climate change in Mediterranean mountains**

Luca Di Nuzzo^1**^, Chiara Vallese^2**^, Renato Benesperi^1^, Paolo Giordani^3*^, Alessandro Chiarucci^2^, Valter Di Cecco^4^, Luciano Di Martino^4^, Michele Di Musciano^5,2^, Gabriele Gheza^2^, Chiara Lelli^2^, Daniel Spitale^6^, Juri Nascimbene^2^

^1^ Università di Firenze, Dipartimento di Biologia, Via la Pira 4, 50121 Firenze, Italia

^2^ Biodiversity & Macroecology Group, Department of Biological, Geological and Environmental Sciences, Alma Mater Studiorum - University of Bologna, Via Irnerio 42, 40126, Bologna, Italy

^3^ Università di Genova, Dipartimento di Farmacia, viale Cembrano, 4, 16148, Genova, Italia

^4^ Parco Nazionale della Majella, Via Badia, 28, 67039 Sulmona, Italia

^5^ University of L'Aquila, Department of Life, Health and Environmental Sciences, Piazzale Salvatore Tommasi 1, 67100 L'Aquila

^6^ Museo di Scienze Naturali dell'Alto Adige, Via Bottai, 1, 39100, Bolzano, Italia

* Corresponding author: Paolo Giordani, Università di Genova, Dipartimento di Farmacia, viale Cembrano, 4, 16148, Genova, Italia, Italia; email: giordani@difar.unige.it

** These authors contributed equally to this work

**Supplementary Table S1.** Lichen species found along the transect, with functional traits and ecological guild. Abbreviations: 1) Growth Form: Sq = Squamulose, Fr = Fruticose, Cr = Crustose, Fo = Foliose. 2) Photobiont: 1) Ch = Chlorococcoid, 2) Cy = Cyanobacteria. 3) Reproduction: S = Sexual reproduction, As = Asexual reproduction. Functional traits were always detectable in specimens identified at genus level. For this latter, temperature-affinities groups were assigned on the basis of the most likely species.

| **Taxon name** | **Growth Form** | **Photobiont** | **Reproduction** | **Temperature-affinities**  **groups** |
| --- | --- | --- | --- | --- |
| *Agonimia tristicula* (Nyl.) Zahlbr. | Sq | Ch | S | Intermediate |
| *Allocetraria madreporiformis* (Ach.) Kärnefelt & A. Thell | Fr | Ch | As | Cryophilous |
| *Amandinea punctata* (Hoffm.) Coppins & Scheid. | Cr | Ch | S | Thermophilous |
| *Arthrorhaphis citrinella (Ach.) Poelt* | Cr | Ch | S | Cryophilous |
| *Athallia saxifragarum* (Poelt) Arup, Frödén & Søchting | Cr | Ch | S | Cryophilous |
| Bacidia sp. | Cr | Ch | S | Thermophilous |
| *Bilimbia lobulata* (Sommerf.) Hafellner & Coppins | Cr | Ch | S | Intermediate |
| *Bilimbia microcarpa* (Th. Fr.) Th. Fr. | Cr | Ch | S | Cryophilous |
| *Blastenia ferruginea* (Huds.) A. Massal. | Cr | Ch | S | Thermophilous |
| *Blastenia herbidella* (Hue) Servít | Cr | Ch | S | Intermediate |
| *Bryoplaca sinapisperma* (DC.) Søchting, Frödén & Arup | Cr | Ch | S | Cryophilous |
| *Caloplaca cerina* (Hedw.) Th. Fr. s.lat. | Cr | Ch | S | Thermophilous |
| Caloplaca sp. | Cr | Ch | S | Thermophilous |
| *Caloplaca stillicidiorum* (Vahl) Lynge | Cr | Ch | S | Cryophilous |
| *Caloplaca stillicidiorum* (Vahl) Lynge var. *muscorum* (A. Massal.) | Cr | Ch | S | Intermediate |
| *Caloplaca subathallina* H. Magn. | Cr | Ch | S | Intermediate |
| *Candelariella commutata* Otte & M. Westb. | Cr | Ch | S | Cryophilous |
| *Candelariella xanthostigma* (Ach.) Lettau | Cr | Ch | S | Thermophilous |
| *Catapyrenium cinereum* (Pers.) Körb. | Sq | Ch | S | Cryophilous |
| *Catapyrenium daedaleum* (Kremp.) Stein | Sq | Ch | S | Cryophilous |
| *Cetraria aculeata* (Schreb.) Fr. | Fr | Ch | As | Intermediate |
| *Cetraria ericetorum* Opiz | Fr | Ch | As | Cryophilous |
| *Cetraria islandica* (L.) Ach. subsp. *islandica* | Fr | Ch | As | Intermediate |
| *Circinaria hispida* (Mereschk.) A. Nordin, Savić & Tibell | Fr | Ch | As | Cryophilous |
| *Cladonia cariosa* (Ach.) Spreng. | Sq | Ch | S | Cryophilous |
| *Cladonia fimbriata* (L.) Fr. | Sq | Ch | As | Intermediate |
| *Cladonia furcata* (Huds.) Schrad. subsp. *furcata* | Fr | Ch | S | Intermediate |
| *Cladonia pocillum* (Ach.) Grognot | Sq | Ch | As | Intermediate |
| *Cladonia pyxidata* (L.) Hoffm. | Sq | Ch | As | Intermediate |
| Cladonia sp | Sq | Ch | As | Thermophilous |
| *Cladonia* cfr. *squamosa* Hoffm. var. *squamosa* | Fr | Ch | As | Intermediate |
| *Cladonia symphycarpa* (Flörke) Fr. | Sq | Ch | As | Intermediate |
| *Dacampia hookeri* (Borrer) A. Massal. | Cr | Ch | S | Cryophilous |
| *Enchylium tenax* (Sw.) Gray | Fo | Cy | S | Intermediate |
| *Flavocetraria nivalis* (L.) Kärnefelt & A. Thell | Fr | Ch | As | Cryophilous |
| Fuscopannaria sp. | Cr | Ch | As | Thermophilous |
| *Gyalolechia bracteata* (Hoffm.) A. Massal. | Cr | Ch | S | Cryophilous |
| Heppia sp. | Cr | Cy | S | Thermophilous |
| *Lathagrium cristatum* (L.) Otálora, P.M. Jørg. & Wedin | Fo | Cy | S | Intermediate |
| *Lathagrium undulatum* (Flot.) Poetsch | Fo | Cy | S | Intermediate |
| *Lecania cyrtella* (Ach.) Th. Fr. | Cr | Ch | S | Intermediate |
| *Lecania sp.* | Cr | Ch | S | Thermophilous |
| *Lecanora chlarotera* Nyl. subsp. *chlarotera* | Cr | Ch | S | Thermophilous |
| *Lecanora epibryon (*Ach.) Ach. var. *epibryon* | Cr | Ch | S | Cryophilous |
| Lecanora sp. | Cr | Ch | S | Thermophilous |
| *Lecidella elaeochroma* (Ach.) M. Choisy var. *elaeochroma* f. *elaeochroma* | Cr | Ch | S | Intermediate |
| *Lecidella euphorea (*Flörke) Hertel | Cr | Ch | S | Intermediate |
| Lecidella wulfenii (Hepp) Körb. | Cr | Ch | S | Cryophilous |
| *Lepraria eburnea* J.R. Laundon | Cr | Ch | As | Thermophilous |
| *Leptogium saturninum* (Dicks.) Nyl. | Fo | Cy | As | Intermediate |
| *Megaspora verrucosa* (Ach.) Arcadia & A. Nordin | Cr | Ch | S | Cryophilous |
| Micarea sp. | Cr | Ch | S | Thermophilous |
| *Myriolecis zosterae* (Ach.) Śliwa, Zhao Xin & Lumbsch var. *palanderi* (Vain.) Śliwa | Cr | Ch | S | Cryophilous |
| *Parvoplaca tiroliensis* (Zahlbr.) Arup, Søchting & Frödén | Cr | Ch | S | Cryophilous |
| *Peltigera* cfr. *malacea* (Ach.) Funck | Fo | Ch | As | Cryophilous |
| *Peltigera rufescens* (Weiss) Humb. | Fo | Cy | As | Intermediate |
| Peltigera sp. | Fo | Cy | S | Intermediate |
| *Physconia muscigena* (Ach.) Poelt var. *muscigena* | Fo | Ch | As | Cryophilous |
| *Placidium lachneum* (Ach.) B. de Lesd. | Sq | Ch | S | Cryophilous |
| *Placidium squamulosum* (Ach.) Breuss | Sq | Ch | S | Thermophilous |
| *Placynthiella icmalea* (Ach.) Coppins & P. James | Cr | Ch | As | Intermediate |
| *Psora decipiens* (Hedw.) Hoffm. | Sq | Ch | S | Intermediate |
| *Rinodina roscida* (Sommerf.) Arnold | Cr | Ch | S | Cryophilous |
| *Romjularia lurida* (Ach.) Timdal | Sq | Ch | S | Intermediate |
| *Rostania ceranisca* (Nyl.) Otálora, P.M. Jørg. & Wedin | Fo | Cy | S | Cryophilous |
| *Scytinium imbricatum* (P.M. Jørg.) Otálora, P.M. Jørg. & Wedin | Sq | Cy | As | Cryophilous |
| *Scytinium lichenoides* (L.) Otálora, P.M. Jørg. & Wedin | Sq | Cy | As | Intermediate |
| *Scytinium schraderi* (Ach.) Otálora, PM Jørg. & Wedin | Sq | Cy | As | Thermophilous |
| *Solorina bispora* Nyl. subsp. *bispora* | Fo | Ch | S | Cryophilous |
| *Solorina bispora* subsp. *macrospora* (Harm.) Burgaz & I. Martínez | Fo | Ch | S | Cryophilous |
| *Thalloidima sedifolium* (Scop.) Kistenich, Timdal, Bendiksby & S.Ekman | Sq | Ch | S | Intermediate |
| Polyblastia sp. | Cr | Ch | S | Intermediate |
| *Trapeliopsis gelatinosa* (Flörke) Coppins & P. James | Cr | Ch | S | Thermophilous |
| Verrucaria sp. | Cr | Ch | S | Thermophilous |

**Supplementary Table S2.** Bryophyte species found along the transect, with functional traits and ecological guild. Length are expressed in mm. Abbreviations: Life form: M = Mat, T = Turf, Tu = Tuft, W = Weft, C = Cushion.

| **Taxon name** | **Lenght (mm)** | **Life Forms** | **Ecological Guild** |
| --- | --- | --- | --- |
| *Barbilophozia lycopodioides* (Wallr.) Loeske | 50 | M | Cryophilous |
| *Barbula unguiculata* Hedw. | 25 | T | Thermophilous |
| *Brachytheciastrum collinum* (Schleich. ex Müll.Hal.) Ignatov & Huttunen | 30 | M | Cryophilous |
| *Brachythecium glareosum* (Bruch ex Spruce) Schimp. | 80 | M | Intermediate |
| *Brachythecium salebrosum* (Hoffm. ex F.Weber & D.Mohr) Schimp. | 70 | M | Intermediate |
| *Sciuro-hypnum starkei* (Brid.) Ignatov & Huttunen | 85 | M | Intermediate |
| *Brachytheciastrum velutinum* (Hedw.) Ignatov & Huttunen | 50 | M | Intermediate |
| *Bryoerythrophyllum ferruginascens* (Stirt.) Giacom. | 32 | T | Intermediate |
| *Bryoerythrophyllum recurvirostrum* (Hedw.) P.C.Chen | 28 | T | Thermophilous |
| *Bryum argenteum* Hedw. | 15 | T | Thermophilous |
| *Bryum caespiticium* Hedw. | 10 | T | Thermophilous |
| *Bryum capillare* Hedw. | 50 | T | Intermediate |
| *Bryum elegans* Nees | 40 | Tu | Intermediate |
| *Campyliadelphus chrysophyllus* (Brid.) R.S.Chopra | 50 | W | Thermophilous |
| *Campylium stellatum* (Hedw.) Lange & C.E.O.Jensen | 100 | W | Thermophilous |
| *Campylium protensum* (Brid.) Kindb. | 60 | W | Thermophilous |
| *Ceratodon purpureus* (Hedw.) Brid. | 35 | T | Thermophilous |
| *Didymodon fallax* (Hedw.) R.H.Zander | 15 | T | Intermediate |
| *Distichium capillaceum* (Hedw.) Bruch & Schimp. | 63 | Tu | Cryophilous |
| *Ditrichum flexicaule* (Schwägr.) Hampe | 50 | Tu | Intermediate |
| *Ditrichum gracile* (Mitt.) Kuntze | 110 | T | Thermophilous |
| *Encalypta streptocarpa* Hedw. | 60 | Tu | Intermediate |
| *Encalypta vulgaris* Hedw. | 20 | Tu | Thermophilous |
| *Eurhynchiastrum pulchellum* (Hedw.) Ignatov & Huttunen | 30 | M | Cryophilous |
| *Fissidens pusillus* (Wilson) Milde | 6 | T | Thermophilous |
| *Homalothecium lutescens* (Hedw.) H.Rob. | 100 | W | Intermediate |
| *Hypnum revolutum* (Mitt.) Lindb. | 55 | M | Cryophilous |
| *Lescuraea saxicola* (Schimp.) Molendo | 30 | M | Intermediate |
| *Lophocolea heterophylla* (Schrad.) Dumort. | 20 | M | Intermediate |
| *Mnium thomsonii* Schimp. | 60 | T | Intermediate |
| *Phascum cuspidatum* Hedw. | 10 | Tu | Thermophilous |
| *Pohlia cruda* (Hedw.) Lindb. | 40 | Tu | Cryophilous |
| *Polytrichum juniperinum* Hedw. | 70 | T | Thermophilous |
| *Pseudoleskeella catenulata* (Brid. ex Schrad.) Kindb. | 15 | M | Intermediate |
| *Ptychodium plicatum* (Schleich. ex F.Weber & D.Mohr) Schimp. | 100 | M | Cryophilous |
| *Racomitrium canescens* (Hedw.) Brid. | 80 | T | Cryophilous |
| *Scapania aequiloba* (Schwagr.) Dumort. | 40 | M | Cryophilous |
| *Schistidium atrofuscum* (Schimp.) Limpr. | 30 | C | Intermediate |
| *Schistidium elegantulum* H.H.Blom | 40 | C | Thermophilous |
| *Syntrichia norvegica* F.Weber | 35 | Tu | Cryophilous |
| *Syntrichia ruralis* (Hedw.) F.Weber & D.Mohr | 50 | T | Thermophilous |
| *Timmia austriaca* Hedw. | 90 | T | Cryophilous |
| *Tortella inclinata* var. *densa* (Lorentz & Molendo) Limpr | 40 | Tu | Thermophilous |
| *Tortella fragilis* (Hook. & Wilson) Limpr. | 25 | Tu | Cryophilous |
| *Tortella tortuosa* (Hedw.) Limpr. | 40 | Tu | Thermophilous |
| *Tortula hoppeana* (Schultz) Ochyra | 10 | Tu | Cryophilous |
| *Weissia controversa* Hedw. | 10 | T | Intermediate |

**Supplementary Table S3.** Plant species found along the transect, with functional traits and ecological guild. Maximum Height are expressed in cm. Abbreviations: Life form: H = Hemicryptophyte, Ch = Chamaephyte, G = Geophyte, T = Therophyte, P = Phanerophyte. Functional traits were always detectable in specimens identified at genus level. For this latter, temperature-affinities groups were assigned on the basis of the most likely species.

| **Taxon name** | **Life Forms** | **Maximum Height (cm)** | **Ecological Guild** |
| --- | --- | --- | --- |
| *Achillea barrelieri* (Ten.) Sch. Bip. subsp. *barrelieri* | H | 15 | Cryophilous |
| *Alyssum cuneifolium* Ten. | Ch | 15 | Intermediate |
| *Alyssum diffusum* Ten. subsp. *diffusum* | Ch | 15 | Intermediate |
| *Androsace villosa* L. subsp. *villosa* | Ch | 3 | Intermediate |
| *Androsace vitaliana* (L.) Lapeyr. | Ch | 4 | Cryophilous |
| *Anthyllis montana* subsp. *jacquinii* (Rchb. f.) Rohlena | Ch | 30 | Thermophilous |
| *Anthyllis vulneraria* L. | H | 40 | Thermophilous |
| *Arctostaphylos uva-ursi* (L.) Spreng | Ch | 10 | Intermediate |
| *Arenaria grandiflora* L. subsp. *grandiflora* | Ch | 15 | Thermophilous |
| *Armernia gracilis* Ten. subsp. *majellensis* (Boiss.) Arrigoni | H | 40 | Intermediate |
| *Artemisia eriantha* Ten. | Ch | 6 | Cryophilous |
| *Asperula cynanchica* L. | H | 20 | Thermophilous |
| *Aster alpinus* L. subsp. *alpinus* | H | 15 | Cryophilous |
| *Astragalus depressus* L. subs. *depressus* | H | 5 | Intermediate |
| *Astragalus sempervirens* Lam. | Ch | 40 | Intermediate |
| *Betonica alopecuros* subsp. *divulsa* (Ten.) Bartolucci & Peruzzi | H | 40 | Intermediate |
| *Biscutella laevigata* L. subsp. *laevigata* | H | 45 | Thermophilous |
| *Bistorta vivipara* (L.) Delarbre | G | 35 | Cryophilous |
| *Botrychium lunaria* (L.) Sw. | G | 30 | Intermediate |
| *Brachypodium genuense* (DC.) Roem. & Schult | H | 50 | Thermophilous |
| *Bromopsis erecta* (Huds.) Fourr. subsp. *erecta* | H | 60 | Thermophilous |
| *Bupleurum falcatum* L. subsp. *cernuum* (Nyman) Arcang | H | 80 | Thermophilous |
| *Campanula scheuchzeri* Vill. subsp. *scheuchzeri* | H | 30 | Cryophilous |
| *Carduus defloratus* subsp. *carlinifolius* (Lam.) Ces. | H | 60 | Intermediate |
| *Carex humilis* Leyss. | H | 20 | Thermophilous |
| *Carex kitaibeliana* Degen ex Bech. | H | 30 | Intermediate |
| *Carex macrolepis* DC. | H | 60 | Thermophilous |
| *Carex myosuroides* Vill. | H | 15 | Cryophilous |
| Carex sp | H | 30 | Intermediate |
| *Cephalanthera damasonium* (Mill.) Druce | G | 50 | Thermophilous |
| *Cerastium arvense* L. | H | 40 | Thermophilous |
| Cerastium sp. | Ch | 40 | Intermediate |
| *Cerastium thomasii* Ten. | Ch | 60 | Thermophilous |
| *Cerastium tomentosum* L. | Ch | 40 | Thermophilous |
| *Coronilla vaginalis* Lam. | Ch | 25 | Thermophilous |
| Cuscuta sp. | T | 5 | Thermophilous |
| *Cymbalaria pallida* (Ten.) Wettst. | H | 20 | Thermophilous |
| *Cytisus spinescens* C.Presl | Ch | 40 | Thermophilous |
| *Dianthus deltoides* L. subsp. *deltoides* | H | 30 | Thermophilous |
| *Doronicum columnae* Ten. | G | 40 | Intermediate |
| *Draba aizoides* L. | H | 8 | Cryophilous |
| *Dryas octopetala* L. subsp. *octopetala* | Ch | 12 | Cryophilous |
| *Edraianthus graminifolius* (L.) A. DC. subsp. *graminifolius* | Ch | 8 | Intermediate |
| *Epipactis atrorubens* (Hoffm.) Besser | G | 80 | Thermophilous |
| *Erigeron epiroticus* (Vierh.) Halácsy | H | 8 | Intermediate |
| *Erysimum majellense* Polatschek | H | 22 | Intermediate |
| Euphrasia sp. | T | 15 | Thermophilous |
| Festuca campione a foglie sottili | H | 30 | Thermophilous |
| Festuca gr. ovina | H | 30 | Thermophilous |
| Festuca sp. | H | 30 | Thermophilous |
| *Festuca violacea* subsp. *italica* Foggi, Gr. Rossi & Signorini | H | 30 | Cryophilous |
| *Galium anisophyllon* Vill. | H | 15 | Intermediate |
| *Galium lucidum* All. subsp. *lucidum* | H | 70 | Thermophilous |
| *Galium magellense* Ten. | H | 9 | Cryophilous |
| *Gentiana cruciata* L. subsp. *cruciata* | H | 50 | Thermophilous |
| *Gentiana nivalis* L. | T | 12 | Cryophilous |
| *Gentiana orbicularis* Schur | H | 6 | Cryophilous |
| *Gentiana verna* L. subsp. *verna* | H | 9 | Intermediate |
| *Globularia meridionalis* (Podp.) O. Schwarz | Ch | 15 | Thermophilous |
| *Gymnadenia conopsea* (L.) R.Br. | G | 55 | Intermediate |
| Heliantenum sp | Ch | 25 | Thermophilous |
| *Helianthemum nummularium* subsp. *grandiflorum* (Scop.) Schinz & Thell. | Ch | 40 | Thermophilous |
| *Helianthemum oelandicum* subsp. *alpestre* (Jacq.) Ces. | Ch | 25 | Intermediate |
| *Helianthemum oelandicum* subsp. *incanum* (Willk.) G. López | Ch | 25 | Intermediate |
| *Helictochloa praetutiana* (Parl. ex Arcang.) Bartolucci, F. Conti, Peruzzi & Banfi subsp. *praetutiana* | H | 60 | Intermediate |
| *Helictochloa versicolor* (Vill.) Romero Zarco subsp. *versicolor* | H | 40 | Cryophilous |
| *Hieracium* cfr. *pilosum* Schleich. ex Froel. | H | 30 | Cryophilous |
| Hieracium gr. villosa | H | 40 | Cryophilous |
| *Hippocrepis comosa* L. | H | 20 | Thermophilous |
| *Hypericum richeri* Vill. subsp. *richeri* | H | 40 | Thermophilous |
| *Iberis saxatilis* L. subsp. *saxatilis* | Ch | 15 | Thermophilous |
| *Juniperus communis* L. | P | 30 | Thermophilous |
| *Koeleria splendens* C. Presl | H | 40 | Thermophilous |
| *Leontopodium nivale* (Ten.) Hand.-Mazz. | H | 3 | Cryophilous |
| *Leucanthemum tridactylites* (A. Kern. & Huter ex Porta & Rigo) Huter, Porta & Rigo | H | 30 | Intermediate |
| *Leucopoa dimorpha* (Guss.) H. Scholz & Foggi | H | 70 | Intermediate |
| *Linum alpinum* Jacq. | H | 40 | Intermediate |
| *Lotus corniculatus* subsp. *alpinus* (DC.) Rothm. | H | 40 | Thermophilous |
| *Luzula multiflora* (Ehrh.) Lej. | H | 40 | Intermediate |
| *Mcneillia graminifolia* (Ard.) Dillenb. & Kadereit subsp. *rosanoi* (Ten.) F.Conti, Bartolucci, Iamonico & Del Guacchio | Ch | 20 | Thermophilous |
| *Medicago lupulina* L. | T | 25 | Thermophilous |
| *Myosotis graui* Selvi | H | 12 | Intermediate |
| *Noccaea stylosa* (Ten.) Rchb. | Ch | 6 | Cryophilous |
| *Omalotheca diminuta* (Braun-Blanq.) Bartolucci & Galasso | H | 10 | Cryophilous |
| *Onobrychis viciifolia* Scop | H | 70 | Thermophilous |
| *Orobanche gracilis* Sm. | T | 50 | Intermediate |
| *Oxytropis campestris* (L.) DC. | H | 20 | Intermediate |
| *Parnassia palustris* L.subsp. *palustris* | H | 30 | Intermediate |
| *Paronychia kapela* (Hacq.) A. Kern. subsp. *kapela* | H | 8 | Intermediate |
| *Pedicularis elegans* Ten. | H | 12 | Intermediate |
| *Petrosedum rupestre* (L.) P.V.Heath | Ch | 30 | Thermophilous |
| *Phyteuma orbiculare* L. | H | 50 | Intermediate |
| *Pilosella officinarum* Vaill. | H | 25 | Intermediate |
| *Pinus mugo* Turra subsp. *mugo* | P | 500 | Intermediate |
| *Plantago atrata* Hoppe | H | 10 | Intermediate |
| *Plantago lanceolata* L. | H | 50 | Thermophilous |
| *Plantago media* L. subsp. *media* | H | 60 | Thermophilous |
| *Poa alpina* L. subsp. *alpina* | H | 50 | Intermediate |
| *Polygala alpestris* subsp. *angelisii* (Ten.) Nyman | H | 10 | Cryophilous |
| *Potentilla crantzii (*Crantz) Beck ex Fritsch | H | 10 | Cryophilous |
| *Potentilla rigoana* Th. Wolf | H | 20 | Thermophilous |
| *Pulsatilla alpina* subsp. *millefoliata* (Bertol.) D.M. Moser | H | 50 | Intermediate |
| *Pyrola minor* L. | H | 30 | Intermediate |
| *Ranunculus brevifolius* Ten. | G | 10 | Intermediate |
| Ranunculus gr. montanus | H | 40 | Intermediate |
| Ranunculus sp. | H | 40 | Intermediate |
| *Rhinanthus wettsteinii* (Sterneck) Soó | T | 50 | Intermediate |
| *Robertia taraxacoides* (Loisel.) DC. | H | 15 | Thermophilous |
| *Rumex acetosa* L. subsp. *acetosa* | H | 110 | Thermophilous |
| *Sabulina verna* (L.) Rchb. subsp. *verna* | Ch | 12 | Thermophilous |
| *Salix retusa* L. | Ch | 40 | Cryophilous |
| *Saxifraga oppositifolia* L. subsp. *speciosa* (Dörfl. & Hayek) Engl. & Irmsch. | Ch | 6 | Cryophilous |
| *Saxifraga paniculata* Mill. | H | 40 | Cryophilous |
| Saxifraga sp. | H | 40 | Intermediate |
| *Scabiosa columbaria* L. | T | 40 | Thermophilous |
| *Scorzoneroides montana* (Lam.) Holub subsp. *breviscapa* (DC.) Greuter | H | 15 | Cryophilous |
| *Sedum atratum* L. | T | 4 | Cryophilous |
| *Sedum hispanicum* L | T | 8 | Thermophilous |
| *Sedum magellense* Ten. | Ch | 15 | Intermediate |
| *Sempervivum arachnoideum* L. | Ch | 10 | Intermediate |
| *Senecio doronicum* subsp. *orientalis* J. Calvo | H | 70 | Cryophilous |
| *Seseli tommasinii* Rchb.f. | H | 100 | Thermophilous |
| *Sesleria juncifolia* Wulfen ex Suffren subsp. *juncifolia* | H | 40 | Intermediate |
| *Sesleria nitida* Ten. | H | 70 | Thermophilous |
| *Silene acaulis* subsp. *bryoides* (Jord.) Nyman | Ch | 5 | Cryophilous |
| *Silene ciliata* Pourr. subsp. *graefferi* (Guss.) Nyman | H | 50 | Intermediate |
| *Sorbus aria* (L.) Crantz | P | 2000 | Thermophilous |
| *Taraxacum apenninum* (Ten.) DC. | H | 15 | Cryophilous |
| *Taraxacum glaciale* É. Huet & A. Huet ex Hand.-Mazz. | H | 15 | Cryophilous |
| *Teucrium montanum* L. | Ch | 15 | Thermophilous |
| Thymus sp. | Ch | 6 | Thermophilous |
| *Tifolium repens* L. | Ch | 20 | Thermophilous |
| *Trifolium montanum* subsp. *rupestre* (Ten.) Nyman | H | 50 | Intermediate |
| *Trifolium noricum* Wulfen | H | 20 | Intermediate |
| *Trifolium pratense* L. sl | Ch | 40 | Thermophilous |
| *Trifolium pratense* L. subsp. *semipurpureum* (Strobl) Pignatti | Ch | 40 | Thermophilous |
| *Trifolium pratense* subsp. *nivale* Ces. | Ch | 40 | Intermediate |
| Trifolium sp. | Ch | 40 | Intermediate |
| *Trifolium thalii* Vill. | H | 15 | Cryophilous |
| *Trinia dalechampii* (Ten.) Janch. | H | 12 | Intermediate |
| *Valeriana montana* L. | H | 40 | Intermediate |
| *Valeriana saliunca* All. | H | 6 | Cryophilous |
| Verbascum sp. | H | 120 | Thermophilous |
| *Veronica aphylla* L. subsp. *aphylla* | H | 8 | Cryophilous |
| *Viola eugeniae* Parl. subsp. *eugeniae* | H | 8 | Intermediate |
| *Ziziphora granatensis* subsp. *alpina* (L.) Brauchler & Gutermann | Ch | 30 | Intermediate |
